# Stem Cell Theory of Cancer: Origin of Tumor Heterogeneity and Plasticity

**DOI:** 10.3390/cancers13164006

**Published:** 2021-08-09

**Authors:** Shi-Ming Tu, Miao Zhang, Christopher G. Wood, Louis L. Pisters

**Affiliations:** 1 Department of Genitourinary Medical Oncology, The University of Texas MD Anderson Cancer Center, Houston, TX 77030, USA; 2 Department of Pathology, The University of Texas MD Anderson Cancer Center, Houston, TX 77030, USA; MZhang8@mdanderson.org; 3 Department of Urology, The University of Texas MD Anderson Cancer Center, Houston, TX 77030, USA; cgwood@mdanderson.org (C.G.W.); lpisters@mdanderson.org (L.L.P.)

**Keywords:** tumor heterogeneity, cancer subtypes, cancer stem cells, unified theory, clonal origin, precision medicine, targeted therapy

## Abstract

**Simple Summary:**

Tumor heterogeneity complicates our diagnoses, confounds our prognoses, and challenges our therapies. Unless we understand the origin of tumor heterogeneity, our diagnosis of cancer will be unsatisfactory, our prognosis uncertain, and treatment unreliable. We observe heterogeneity in myriad mixed tumors, including testicular, lung, and breast cancers. We recognize heterogeneity in diverse tumor subtypes, no matter how we subgroup and subdivide them. We postulate that cancer subtypes can be meaningless and useless without a proper theory about cancer’s stem cell versus genetic origins. We propose that tumor heterogeneity alludes to a stem cell theory of cancer and provides clues that cancer is a stem cell disease. A stem cell, as opposed to a genetic, origin of cancer constitutes a unified theory of cancer, which predicates that the same genetic abnormalities and microenvironmental aberrations lead to different biological effects and clinical outcomes in a progenitor stem cell versus a mature progeny cell.

**Abstract:**

In many respects, heterogeneity is one of the most striking revelations and common manifestations of a stem cell origin of cancer. We observe heterogeneity in myriad mixed tumors including testicular, lung, and breast cancers. We recognize heterogeneity in diverse tumor subtypes in prostate and kidney cancers. From this perspective, we illustrate that one of the main stem-ness characteristics, i.e., the ability to differentiate into diverse and multiple lineages, is central to tumor heterogeneity. We postulate that cancer subtypes can be meaningless and useless without a proper theory about cancer’s stem cell versus genetic origin and nature. We propose a unified theory of cancer in which the same genetic abnormalities, epigenetic defects, and microenvironmental aberrations cause different effects and lead to different outcomes in a progenitor stem cell versus a mature progeny cell. We need to recognize that an all-encompassing genetic theory of cancer may be incomplete and obsolete. A stem cell theory of cancer provides greater universality, interconnectivity, and utility. Although genetic defects are pivotal, cellular context is paramount. When it concerns tumor heterogeneity, perhaps we need to revisit the conventional wisdom of precision medicine and revise our current practice of targeted therapy in cancer care.

“*Nature creates unity even in the parts of a whole*”, Eugène Delacroix.

When we treat patients with cancer in the clinic, it is evident that heterogeneity is an intrinsic property of cancer. However, when we study cancer in the laboratory, it is necessary for us to be reductionist—to narrow down variables and simplify parameters. Conceptually, we often prefer to see cancer as a pure rather than mixed entity and treat it as a simple rather than complex malady. However, when we only look at the parts of a whole, we may not discern unity among the small pieces. When we pretend that cancer is homogenous, we may confuse our artificial reductionism with reality.

Tumor heterogeneity complicates our diagnoses, confounds our prognoses, and challenges our therapies [1,2,3]. Unless we understand the origin and nature of tumor heterogeneity, our diagnosis of cancer will be unsatisfactory, our prognosis uncertain, and treatment unreliable.

Ultimately, elucidating the origin of tumor heterogeneity means elaborating a genetic versus stem cell theory of cancer. Do certain genetic mutations cause tumor heterogeneity, or does the cellular context of those genetic mutations play a more critical role in cancer’s protean manifestations?

In this article, we reiterate that heterogeneity is synonymous with plasticity [4,5,6,7,8,9,10]. We postulate that tumor heterogeneity alludes to a unified theory of cancer and provides clues that cancer has a stem-ness origin and is a stem cell disease [11,12]. We propose that the genetic theory of cancer has flaws, especially when it does not account for tumor heterogeneity. Regarding tumor heterogeneity, genetic defects may be king, but cellular context is key.

## 1. Baskets and Umbrellas

If there is a universal truth about cancer, then there should be a common thread that unites its various properties. This universal truth will enable us to envision cancer as a single entity—as befitting a single “basket”—which simplifies our conception, articulation, and therapeutics of cancer.

In many respects, the stem cell theory of cancer embraces this “basket” criterion. If cancer has a stem cell origin and displays stem-ness characteristics, such as self-renewal and asymmetric division, then a strategy to treat malignant stem cells and their stem-like microenvironment would be relevant and applicable to cancers as diverse as leukemia, melanoma, and hepatoma.

In contrast, the obvious complexity of cancer suggests that there must be categories and subcategories of cancer with unique identities and characteristics. This observation encourages us to divide cancer into separate groups—as in “umbrellas”—which simplifies our narrative of cancer in a different way.

Paradoxically, the stem cell theory of cancer also encompasses this “umbrella” criterion. When all cancers have a stem-ness origin but are derived from discrete progenitor cells in a stem cell hierarchy with disparate potency, they belong to different cancer subtypes with their own specific genetic signatures and epigenetic profiles, such as seminomas versus non-seminomas, small cell versus non-small cell lung cancers, and ductal versus lobular breast cancers.

## 2. Clonal Origin

Although genetic markers are invaluable for the purposes of tracing a clonal origin and tracking the cellular lineages of cancer [13,14], we need to be wary of whether the markers themselves are actually the cause or merely an effect of cancer.

Fortunately, various mixed tumors provide invaluable opportunities to investigate and elucidate their clonal origin and derivation.

### 2.1. Testicular Cancer

About half of germ cell tumors of the testis (TGCT) are seminomas, and the other half are non-seminomas. Approximately 80% of non-seminomas are mixed tumors comprising embryonal carcinoma, choriocarcinoma, yolk sac tumors, teratoma, and/or seminoma in more than 30 different combinations, permutations, and proportions [15].

Importantly, TGCT is a preeminently curable cancer (>90% cure rate) even when it is advanced and metastatic, in part because we know how to deal with its genotypes and how to treat its phenotypes [15,16,17]. Hence, in a mixed TGCT containing embryonal carcinoma and teratoma, both have the same genetic marker (i.e., i(12p)) and a similar if not identical genetic makeup due to their common clonal origin. However, the former is fulminant and chemo-sensitive, while the latter is indolent and chemo-resistant. To cure a mixed TGCT containing embryonal carcinoma and teratoma, we need to eliminate its systemic component (i.e., embryonal carcinoma) with chemotherapy and eradicate any residual localized tumor (i.e., teratoma) with surgery. Either chemotherapy or surgery alone may not be adequate to cure a mixed TGCT. Targeting genetic defects present in both is likely to be moot, if not futile.

### 2.2. Breast Cancer

One may categorize breast cancer into the invasive ductal (80%) and lobular (about 15%) subgroups. However, one cannot help but also recognize a mixed ductal-lobular (MDL) subgroup (up to 5%), defined as having a ductal component constituting at least 10% of the tumor and a lobular component of at least 50% [18].

In addition, one can further subdivide both invasive ductal and lobular breast cancers into luminal, basal, and HER-2-like subtypes based on their expression of estrogen (ER), progesterone (PR), and HER-2 receptors on the tumor cells. Although lobular breast cancer is predominantly luminal (ER^+^, PR^+^), a small fraction may be basal (or triple negative: ER^-^, PR^-^, and HER-2^−^) or HER-2-like, not unlike its ductal counterpart.

McCart–Reed et al. performed comparative genomic hybridization and multi-region whole-exome sequencing of four representative cases of MDL breast carcinoma [18]. They found a common clonal relationship between all morphologically distinct components within individual cases. However, the mutations varied between cases. Their results support a model in which separate morphological components of MDL arise from a common progenitor cell, and a lobular component can arise from ductal morphology. In MDLs that present with lobular carcinoma in situ (LCIS) and ductal carcinoma in situ (DCIS), clonal divergence occurs early and is frequently associated with complete loss of E-cadherin expression. Meanwhile, in the majority of MDLs, which present with DCIS but not LCIS, clonal divergence from the ductal to the lobular phenotype occurs late in tumor evolution and is associated with aberrant E-cadherin expression.

One pertinent question pertaining to a genetic versus stem cell origin is whether E-cadherin is the real maker or a mere marker of MDL breast cancer. Does it cause or is it just an effect of the evolution of MDL breast cancer? 

### 2.3. Lung Cancer

Similarly, one can classify lung cancer into the small cell (SCLC) (15%) and non-small cell (NSCLC) (85%) subgroups. However, about 10% of these patients have combined or mixed SCLC and NSCLC (MSN) [19,20].

To make matters more complicated, NSCLC by itself is a diverse group with distinct histological phenotypes, which include squamous cell carcinoma, adenocarcinoma, and large cell carcinoma. Mutations in EGFR (up to 35%) tend to occur in adenocarcinomas, but may also be present in squamous cell carcinomas and less commonly in SCLC.

Patients with MSN have decreased overall survival compared with those with pure SCLC. Approximately 75% of the identified somatic mutations are present in both components. These findings suggest a common precursor with subsequent acquisition of oncogenic changes in MSN.

One wonders whether additional mutations render MSN more deadly, even though the mutations seem to occur in a sporadic and random fashion. One also wonders whether it is the genetic makeup or the cell of origin that determines the prognostic value and the predictive power of our current diagnostic capabilities and therapeutic options.

## 3. Genetics vs. Epigenetics

It is evident that epigenetic rather than genetic changes in a tumor are another representation, perhaps a better one, of what constitutes cancer [10,21,22]. Epigenetics captures dynamic and interactive processes in a tumor. It also encapsulates the expression and exhibition of its underlying heterogeneous, dormant, and immune-evasive capabilities and nature.

After all, the genetic blueprint of all the diverse cells in our bodies is almost the same, if not completely identical. It is the epigenetic output that makes cells different, whether they are branded or generic, working or resting, protean or immutable.

What is not obvious is that the epigenetic output of our cells may provide the clue to a cellular rather than a genetic origin of cancer. When the epigenome trumps the genome, we see a more detailed and complete molecular profile that alludes to a stem cell origin of cancer. The epigenetics of cancer illustrates that carcinogenesis mirrors embryogenesis—in other words, that oncology recapitulates ontogeny—and that all cancer hallmarks are also innate stem-ness earmarks.

For example, genetic biomarkers, such as loss of *PRBM1* or *BAP1*, may be useful for the identification of specific kidney tumor subtypes with distinct prognostic and/or predictive implications [23,24,25]. Hence, loss of *PBRM1* (50% of clear cell renal cell carcinomas (ccRCCs)) enhances proangiogenic activity in the microenvironment, with favorable effects on the response to tyrosine kinase inhibitors (TKIs), while *BAP1* loss (15% of ccRCCs) correlates with decreased angiogenic signaling and adverse outcomes to TKIs. Although *PBRM1* and *BAP1* seem to be mutually exclusive, they are both located on chromosome 3p. *PBRM1* encodes BAF180 and is involved in the SWI/SNF family of remodelers, while *BAP1* is integral to the Polycomb group’s (PcG) functions in cell fate determination in early development. Therefore, both *PBRM1* and *BAP1* affect stem cell function in one way or another.

Importantly, *PRMB1* is also an epigenetic biomarker, because it affects the epigenetic functions of a progenitor stem cell or a progeny differentiated cell in different ways [26]. Clearly, when it concerns *PRBM1*, we need to look beyond genetics and examine the epigenetics of the involved cells, e.g., how they affect or are affected by their respective microenvironments.

In many respects, *PRBM1*-deficient or -loss cells are equivalent to *PRBM1*-negative or -inactive cells. Because *PRBM1*-negative or -inactive cells are essentially mature or differentiated cells, *PRBM1*-deficient or -loss tumors are likely to be well-differentiated rather than poorly differentiated tumors. Because well-differentiated tumors tend to have a protracted and indolent natural history, *PRBM1*-deficient/loss cancers should impart a relatively favorable clinical (i.e., prognostic) outcome, even without treatment. Because differentiated tumors may be amenable to specific treatment modalities, *PRBM1*-deficient/loss cancers would also elicit a superior clinical (i.e., predictive) response with certain therapeutic interventions.

## 4. Genomic Subtypes

One way to solve the problem of tumor heterogeneity is to categorize tumors into convenient, perhaps contrived, tumor subtypes. How we create and divide tumors into subgroups and whether those subgroups are meaningful and useful depends on whether we believe in a genetic versus stem cell origin of cancers.

Take prostate cancer as an example. Does genomic subtyping of prostate cancer improve the prognostic value and enhance the predictive power of our current diagnostic capabilities and therapeutic options?

The Cancer Genome Atlas Research Network verified molecular heterogeneity in primary prostate cancer [27]. Notably, 74% of primary prostate cancer could be classified into seven subtypes, as defined by specific gene fusions (*ERG* [46%], *ETV1* [8%], *ETV4* [4%], and *FLI1* [1%]) or mutations (*SPOP* [11%], *FOXA1* [3%], and *IDH1* [1%]). Remarkably, 53% had ETS family gene fusions (i.e., *ERG*, *ETV1*, *ETV4*, *FLI1*). However, individuals with or without fusion-bearing tumors have a similar prognosis following prostatectomy [28,29]. Importantly, there is significant diversity in DNA copy-number alterations, gene expression, and DNA methylation within the subgroups, as well as significant overlap in copy number alterations and mutations among the subgroups.

In other words, despite our valiant attempt to separate tumors into pertinent subtypes using sophisticated genomic tools and technology, the tumors appear to sabotage this effort by sharing genetic biomarkers that exhibit a striking similarity in their distribution among the purported subtypes.

Van Dessel et al. performed whole-genome analysis of fresh frozen biopsies from 197 metastatic castration-resistant prostate cancer (CRPC) cases [30]. They defined eight distinct genomic clusters, including clinically relevant genotypes, e.g., 7% with microsatellite instability for which immunotherapy may be beneficial [31], as well as 7% with *CDK12*-/- and 11% with homologous recombination deficiency enriched with genomic deletions and *BRCA2* aberrations for which a PARP inhibitor [32] may be effective. However, a majority of metastatic CRPC (65.5%) did not fall into any as-yet clinically relevant or biologically apparent genotypes that would implicate a role for targeted therapy.

Interestingly, Van Dessel et al. did not find any striking primary-only genomic subgroups, nor did they detect the presence of the metastatic CRPC-derived genomic subgroups in the primary prostate cancer cohort [30]. Again, overlap among the subgroups and instability of the results over time suggest that genotype grouping could be an artifact and might not be as clinically meaningful or useful as we expect or wish.

## 5. Stem-Ness Subtypes

In contrast, when we categorize prostate cancer based on stem-ness signatures rather than genomic changes, perhaps we have a better chance to improve the prognostic value and enhance the predictive power of our current diagnostic capabilities and therapeutic options.

Smith et al. demonstrated that aggressive prostate cancer shares a conservative transcriptional program with normal adult basal stem cells [33,34]. They showed that a set of E2F target genes is common between prostate small cell neuroendocrine carcinoma and primary prostate basal cells. Labrecque et al. performed rapid autopsy studies, whole-genome sequencing, gene set enrichment analysis, and immunohistochemistry studies in 98 metastatic CRPC cases and confirmed small cell or neuroendocrine expression without androgen receptor (AR) activity in one of the lethal subgroups [35]. Indeed, they reaffirmed that metastatic CRPC is a disease of continuum, rather than of category. Some subtypes may convert into other subtypes. CRPC is fluid, not static.

Aggarwal et al. reported that 17% of patients heavily treated with chemotherapy and enzalutamide developed “treatment-emergent neuroendocrine prostate cancer” [36]. More recently, Alumkal et al. identified an AR activity-low, stem-like program associated with enzalutamide resistance [37]. Importantly, they found no statistical difference between the sensitive and resistant groups with respect to *TP53*, *RB1*, *PTEN*, or other gene defects. Neither alterations in the AR gene nor AR expression correlated with de novo enzalutamide resistance. Epithelial-to-mesenchymal transition (EMT) was the top gene set activated in non-responders. Non-responders had other activated pathways linked to EMT, including IL-6/Jak/Stat3, TGF-beta, and TNF-alpha/NFkB signal pathways. Non-responders had activated basal lineage and neurogenic/stem-like programs, while responders showed activated luminal lineage programs. In addition, low AR transcriptional activity, rather than AR genomic changes or altered AR expression, contributed to de novo enzalutamide resistance.

The results from Aggarwal’s and Alumkal’s studies [36,37] support a stem cell theory of cancer, in which cellular context rather than genetic aberration is central to carcinogenesis. When a stem cell origin of cancer embraces and encompasses a whole system of various pathways and entire cellular networks, it is a unified theory of cancer.

If lethal CRPC subtypes, such as anaplastic variants with neuroendocrine features, have EMT phenotypes with stem-like properties, then it is imperative that we keep them in mind in all therapeutic considerations. Formulation of a relevant stem cell versus genetic theory of cancer will enable the design of clinical trials and the selection of appropriate cancer patients, as well as the proper stratification of tumor subtypes in the right way at the right time. This will ensure that we pursue therapy, rather than mere drug development [38], to solve the challenges of tumor heterogeneity, e.g., treating both AR+, PSA-producing, differentiated progeny cells and AR-, non-PSA producing, stem-like progenitor cells.

## 6. Renal Cell Carcinoma (RCC) Subtypes

When it concerns a genetic or a stem cell origin of tumor heterogeneity, RCC provides another illustrative tumor model.

Brannon et al. speculated that heterogeneity in ccRCC could arise when *VHL* mutation, HIF stabilization, and secondary mutations occur during multiple-step carcinogenesis [39].

Alternatively, it is also plausible that the cell of origin in which *VHL* mutation, HIF stabilization, and secondary mutations occur (rather than the molecular defects themselves) determines heterogeneity in ccRCC.

According to the genetic theory of cancer, *VHL* may be the driver, the cause, and the maker of RCC. In contrast, a stem cell theory of cancer predicates that VHL is a passenger, an effect, and a marker of ccRCC.

When cellular context is paramount, whether genetic defects such as *VHL* mutation are present in a progenitor stem-like cell or progeny differentiated cell will determine the tumor subtype, the malignant potential, and the clinical outcome.

Hence, we observe genetic defects including *VHL* mutations both in malignant RCC and in benign tumors and nonmalignant tissues. Brannon et al. found that *VHL* mutations did not differentiate two distinct subtypes of RCC (ccA and ccB), which displayed different gene expression profiles and clinical outcomes [39]. Similarly, Dahinden et al. found that pVHL and phosphor-mTOR staining inversely correlated with tumor stage and grade but did not correlate with survival [40].

In contrast, stem-like phenotypes, unlike genetic markers, did affect clinical outcomes. Brannon et al. showed that RCC ccB originated from earlier progenitor cells (associated with EMT, TGF-beta, Wnt, and wound-healing gene overexpression), while RCC ccA derived from later progenitor cells (associated with genes involved in angiogenesis and fatty acid metabolism). The former subtype predicts worse cancer-specific survival compared with the latter (2.0 years versus 8.6 years [*p* = 0.0002], respectively).

Interestingly, Zhao et al. published similar results that validated these findings [41]. RCC subgroups whose gene expression profiles resembled that of the terminal differentiated normal renal cortex (i.e., derived from later progenitor cells in a stem cell hierarchy) elicited better prognosis compared with another RCC subgroup whose gene expression profile reflected wound healing (i.e., associated with loss of differentiation and gain of EMT, and derived from an earlier progenitor cell in a stem cell hierarchy) which fared worse.

## 7. Evolutionary Subtypes

In theory, genomics should be informative and useful in cancer medicine; in practice, it can be uninformative and useless. Turajlic et al. demonstrated that up to 30 mutational and somatic copy number alteration (SCNA) driver events are detectable in a single RCC tumor [42]. For larger tumors, four to eight biopsies are required to capture most of these events. The gain in driver detection per additional biopsy begins to decline after about eight biopsies.

Nevertheless, they identified seven distinct evolutionary RCC subtypes to address a fundamental question in cancer biology: which patients tend to harbor widespread occult metastases and may not benefit from upfront surgery [43].

They discovered that primary tumors with low intratumoral heterogeneity (ITH) but elevated SCNAs had rapid progression at multiple sites. Consequently, patients with such tumors are unlikely to benefit from surgery. They may not respond to immunotherapy or TKIs either.

Those with high ITH had an attenuated pattern of progression. They are associated with increased risk of metastasis, but not with decreased survival. Patients with such tumors may still benefit from surgery, have PDL1 expression, and respond to immunotherapy.

Those with low ITH and a low fraction of the genome affected by SCNAs had overall low metastatic potential. Patients with such tumors benefit from surgery, have an angiogenesis profile, and respond to TKIs.

Contrary to the multistep model of carcinogenesis and a genetic origin of cancer [44], Turajlic et al. showed that metastatic lesions tend to harbor fewer driver somatic alterations (mean: 9) compared to their matched primary tumors (mean: 12) [43].

The results from their study suggest that removal of the primary tumor is beneficial for those high-ITH, indolent RCC subtypes (even with metastatic disease), but not for the low-ITH, high-SCNA, fulminant ones (without evidence of metastatic disease). Interestingly, low ITH and high SCNAs (unlike SNV/INDEL) reflect aneuploidy and implicate aberrant asymmetric division, suggesting that a stem-ness origin may be involved in the evolution of this malignant phenotype. Intriguingly, evidence of immune evasion also suggests a stem-ness origin in this RCC subtype.

## 8. Hierarchy vs. Stochasticity

When we aspire to elucidate a stem cell versus genetic origin of tumor heterogeneity, we may find a proper perspective on hierarchy versus stochasticity illuminating and a pertinent narrative on differentiation versus dedifferentiation enlightening.

**Figure 1:**
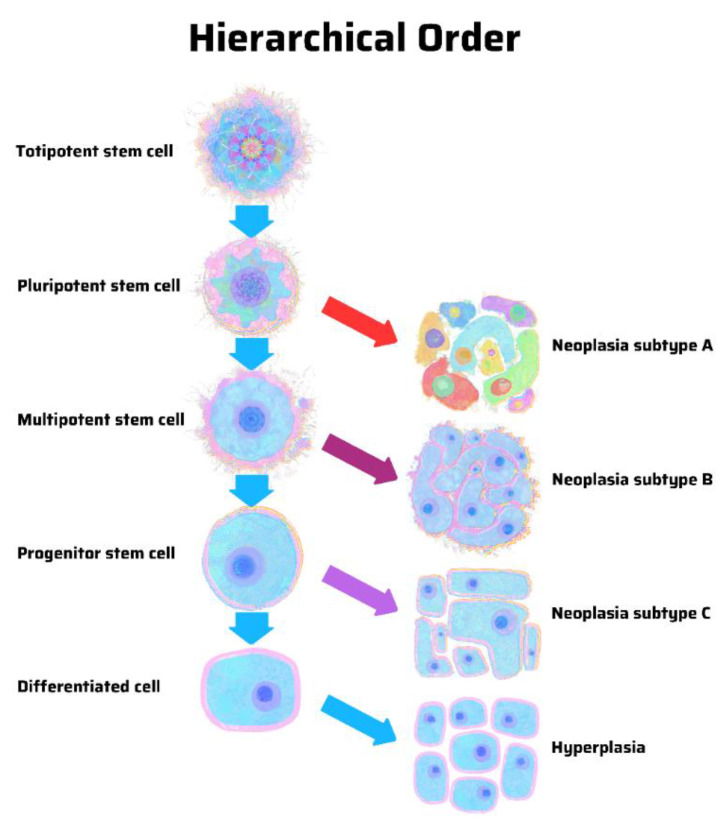
Origin of cancer in a hierarchical order, according to the stem cell theory of cancer. Unique cancer subtypes arise from distinct progenitor cancer-initiating cells with tapering stem-ness properties. In a differentiated cell of stem cell origin, the same genetic mutations may cause hyperplasia rather than neoplasia. Illustration by Benjamin Tu. Adapted with permission from Nova Publishers [12].

**Figure 2:**
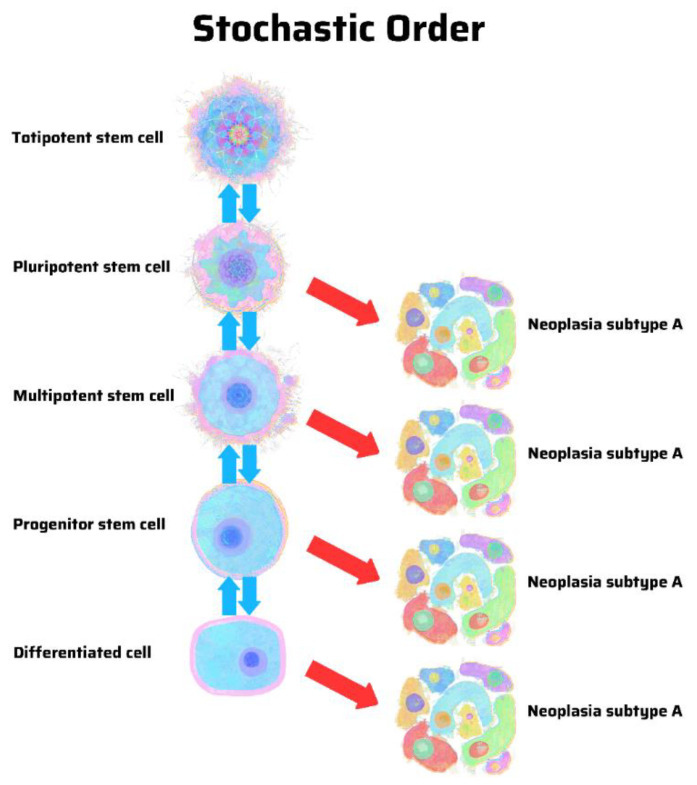
Origin of cancer in a stochastic order, according to the genetic theory of cancer. The same genetic mutations may cause similar cancer subtypes without regard for their cellular origins, including those in a differentiated cell of stem cell origin. Illustration by Benjamin Tu. Adapted with permission from Nova Publishers [12].

The hypothesis of a stem cell origin of cancer predicates that there is a hierarchy of progenitor cancer cells analogous to that in normal stem cell development. In contrast, the hypothesis of dedifferentiation in cancer argues for a stochastic model in which random mutations can cause any cell in the body to develop into a cancer cell. There is a pattern in hierarchy and uncertainty in stochasticity. A hierarchical model predicts that cancer subtypes derive from specific progenitor cells in a hierarchy (Figure 1). In contrast, a stochastic model predicts that cancer can arise from any cell, stem-like or differentiated (Figure 2) [12].

In many respects, stem-ness implicates heterogeneity. A multipotent cell has the capacity to differentiate into multiple cellular lineages and assume various cellular phenotypes. A less potent cell has less capacity to do so. A differentiated cell has no capacity to do so.

However, the idea that cells are plastic and reprogrammable also suggests that they are interchangeable and reversable. As a result of genetic mutations, any cells can be altered, changed, or reversed. When all cells are supposedly malleable or mutable, the situation is more compatible with stochasticity than hierarchy. Nevertheless, one cannot help but notice that there is still a hierarchical order in this stochastic tendency, i.e., those very progenitor cells with greater stem-ness have a greater capacity to be altered, changed, or reversed.

## 9. Differentiation vs. Dedifferentiation

It is conceivable that potency and heterogeneity are somehow interlinked and ingrained in the process of differentiation, as much as in the process of dedifferentiation. However, the difference between differentiation and dedifferentiation cannot be more antithetical, and fundamental to the stem cell versus a genetic origin of cancer.

A stem cell theory of cancer argues for a hierarchical model in which only certain cells in a stem cell hierarchy may develop into distinct tumor subtypes (Figure 1). Classic experiments using mouse strain 129 showed that embryonal carcinoma cells (rather than teratoma cells) isolated from teratocarcinomas underwent self-renewal and differentiation into a wide variety of cell types. Those experiments laid the groundwork for embryonic stem cell (ESC) research [45]. Importantly, they established that embryonal carcinoma cells constitute the abnormal malignant counterparts of ESCs and revealed an uncannily close relationship between cancer cells and stem cells [46].

In contrast, the theory of cancer dedifferentiation argues for a stochastic model in which any cell in the body can develop into a cancer (Figure 2). In many respects, the discovery of induced pluripotent stem cell (iPSC) galvanized this school of thought by demonstrating that the overexpression of certain genes (such as Oct4, Sox2, Klf4, and c-myc) could reprogram mouse embryonic fibroblasts into stem cells [47,48]. Importantly, when malignant cells associated with iPSCs are derived from a mature or differentiated cell endowed with stem-ness features, they actually confirm if not prove a stem cell origin of cancer [49,50,51,52,53]. It is as if we have performed an ultimate experiment without the proper or pertinent hypothesis.

## 10. Maturation Arrest

When we have two contrary scientific theories of differentiation versus dedifferentiation, at odds with respect to the revelation of potency and manifestation of heterogeneity, perhaps a middle ground may shed light on the underlying biological mechanisms and scientific processes.

Sell and Pierce [54] postulated that stem cells undergo maturation arrest during cancer formation. Normally, stem cells express stem-ness factors by default. When it is time to differentiate, differentiation factors replace stem-ness factors. When maturation arrest occurs, the resultant abnormal “halfway” cells may be regarded as reprogrammed somatic cells. However, when differentiation is blocked, it will be difficult to distinguish whether the cells remain undifferentiated or become dedifferentiated.

We propose that another way to reconcile the dilemma of differentiation and dedifferentiation is to discern a critical difference between the hypothesis and the experiment [11,12]. According to the scientific method, we formulate a hypothesis based on pertinent observations of nature. We perform an experiment to test the hypothesis, not to generate one. Consequently, a hypothesis derived from the results of an experiment is inherently flawed—the experiment is artificial by necessity and the results when taken out of context can be misleading and misguided.

Therefore, with regard to differentiation versus dedifferentiation in tumor heterogeneity, it is imperative that we determine the verity of the hypotheses—which ones are based on pertinent observations of nature—and assess the validity of the results obtained from the experiments—which ones may be artefacts—because the hypotheses that the experiments are designed to test may be misconceived and misconstrued.

## 11. Precision Medicine and Targeted Therapy

In many respects, we anticipate that heterogeneity is anathema to precision medicine and targeted therapy. When a tumor is heterogeneously pathological and pathologically heterogeneous, precision medicine and targeted therapies may be unrealistic. Instead, we need to think systemically and ensure that our actions are coordinated.

Brastianos et al. demonstrated that although spatially and temporally separated brain metastases were genetically homogeneous, they were highly divergent from the primary tumor from which they originated and from other metastases that disseminated elsewhere [55].

Perhaps precision medicine and targeted therapies that manage to control brain metastases may prolong longevity and enhance quality of life. However, when other sites of disease are just as threatening and may not be amenable to the same precision medicine and targeted therapy, the overall clinical benefit is likely to be qualified and tempered, especially when the treatments also turn out to be exorbitantly expensive and excessively toxic.

To be optimally effective and beneficial, our treatments should address the entire cancer rather than just parts of the cancer. We should also take care of a patient’s whole wellbeing, not just one part of an ailment, to provide a successful if not curative remedy.

To assume precision and forget heterogeneity seems naïve. To target one part and forfeit the whole seem negligent. Perhaps Henri Matisse is right: precision is not reality. After all, cancer is more dynamic than static. Often enough it is complex rather than simple. In origin and by nature, it is interactive, not isolated, interconnected not separated.

We need to recognize that when it concerns cancer heterogeneity, the conventional wisdom of precision medicine and our current practice of targeted therapy in cancer care may inadvertently defy basic cancer biology and contradict common cancer reality.

## 12. Conclusions

In many respects, heterogeneity is one of the most striking revelations and obvious manifestations of a stem cell origin of cancer. From this perspective, we highlighted that one of the main stem-ness characteristics, namely, plasticity—the ability to differentiate into diverse and multiple lineages—is central to tumor heterogeneity.

We observe heterogeneity in myriad mixed tumors, including testicular, lung, and breast cancers. We recognize heterogeneity in diverse tumor subtypes, no matter how we subcategorize, subgroup, and subdivide them. We postulated that cancer subtypes can be meaningless and useless without a proper theory about cancer’s stem cell versus genetic origin and nature.

When we submit and commit to different cancer theories, we have different perspectives and narratives about the role and impact of the cancer genome versus epigenome. Inevitably, our perspective and narrative determine whether we practice precision medicine versus integrated medicine, targeted therapy versus multimodal therapy, and whether we provide only marginal and incremental rather than substantial and exponential clinical benefits in cancer care.

In the end, a one-basket-carries-all or one-umbrella-covers-all genetic theory of cancer is incomplete and obsolete. Everything we have learned about the genetic theory of cancer is still correct, but we need to understand it in the proper context of a stem cell theory of cancer. A unified stem cell theory of cancer predicates that the same genetic abnormalities, epigenetic defects, and microenvironmental aberrations lead to different biological effects and clinical outcomes in a progenitor stem cell versus progeny mature cell.

Perhaps Delacroix would agree that nature creates unity in the form of cellular context in the parts of a whole cancer, whether those parts are the genome, epigenome, transcriptome, metabolome, et cetera. A stem cell rather than a genetic origin of cancer constitutes a unified theory of cancer that unites rather than divides malignant from benign tumor subtypes, as well as heterogeneous from homogeneous phenotypes.

## Data Availability

No new data were created or analyzed in this study. Data sharing is not applicable to this article.
